# The influence of continuous prenatal exposure to valproic acid on physical, nociceptive, emotional and psychomotor responses during adolescence in mice: Dose-related effects within sexes

**DOI:** 10.3389/fnbeh.2022.982811

**Published:** 2022-09-29

**Authors:** Jelena Podgorac, Slobodan Sekulić, Branka Petković, Gordana Stojadinović, Ljiljana Martać, Vesna Pešić

**Affiliations:** ^1^Department of Neurophysiology, Institute for Biological Research “Siniša Stanković” – National Institute of the Republic of Serbia, University of Belgrade, Belgrade, Serbia; ^2^Faculty of Medicine Novi Sad, University of Novi Sad, Novi Sad, Serbia; ^3^Department of Neurology, Clinical Center of Vojvodina, Novi Sad, Serbia; ^4^Department of Neurobiology, Institute for Biological Research “Siniša Stanković” – National Institute of the Republic of Serbia, University of Belgrade, Belgrade, Serbia

**Keywords:** valproic acid, prenatal exposure, adolescent mice, nociception, anxiety, motor activity

## Abstract

Clinical findings show that the use of valproic acid (VPA) during pregnancy increases the risk of birth defects and autism spectrum disorder in offspring. Although there is a consensus that monitoring of potential long-term outcomes of VPA exposure is needed, especially in undiagnosed individuals, preclinical studies addressing this issue are rare. The present study examined the effects of continuous intrauterine exposure to a wide dose range of VPA (50, 100, 200, and 400 mg/kg/day) on the physical and behavioral response in peripubertal mice as a rodent model of adolescence. Body weight and the hot plate test [on postnatal days (PND) 25 and 32], the elevated plus-maze test (on PND35), and the open field test (on PND40) served to examine physical growth, the supraspinal reflex response to a painful thermal stimulus and conditional learning, anxiety-like/risk-assessment behavior, as well as novelty-induced psychomotor activity, respectively. VPA exposure produced the following responses: (i) a negative effect on body weight, except for the dose of 100 mg/kg/day in both sexes; (ii) an increase in the percentage of animals that responded to the thermal stimulus above the defined cut-off time interval and the response latency in both sexes; (iii) dose-specific changes within sexes in behavior provoked by a novel anxiogenic environment, i.e., in females less anxiety-like/risk-assessment behavior in response to the lowest exposure dose, and in males more pronounced anxiety-like/risk-assessment behavior after exposure to the highest dose and 100 mg/kg/day; (iv) dose-specific changes within sexes in novelty-induced psychomotor activity, i.e., in females a decrease in stereotypy-like activity along with an increase in rearing, and in males a decrease in stereotypy-like activity only. These findings show that continuous intrauterine exposure to VPA produces maladaptive functioning in different behavioral domains in adolescence and that the consequences are delicate to assess as they are dose-related within sexes.

## Introduction

Adolescence is a developmental period second to infancy in terms of the rate of developmental changes that can occur within the brain (Arain et al., 2013), with an imbalance between the limbic and reward systems as a characteristic of adolescent brain development (Steinberg, 2008; Konrad et al., 2013). According to the World Health Organization [WHO] (2021), depression, anxiety and behavioral disorders are among the leading causes of illness and disability in adolescents. Importantly, adolescence brings a particular vulnerability to many social-emotional problems not only in typically developing but in atypically developing children as well, and is indicated as an especially vulnerable time (i.e., the second hit) in the developmental course of autism (Picci and Scherf, 2015). Therefore, it is essential to decipher potential risk factors, including those related to prenatal experiences, which can contribute to the vulnerability of adolescents to mental health problems and impact health and functioning in adulthood (Ormel et al., 2017). Recent findings have revealed that developmental irregularities, impairment of school performance at the early-adolescent age, and cumulative risk of intellectual disability up to 18 years persist in subjects exposed to valproic acid (VPA) during intrauterine development who were not diagnosed with congenital malformations (Christensen et al., 2013; Elkjær et al., 2018; Daugaard et al., 2020) suggesting that close monitoring of the potential long-term outcomes of VPA exposure is needed (Daugaard et al., 2020). However, the relationship between continuous prenatal exposure to VPA and adolescence (in terms of peculiarities in physical development as well as social, emotional and sensory-motor functioning) is still insufficiently investigated.

The VPA is widely used as an antiepileptic drug in the treatment of different types of epilepsy. It is also recognized as a mood stabilizer, improving mood in patients with epilepsy, developmental disabilities, schizoaffective disorder, panic disorder, and borderline personality disorder (Kastner et al., 1993; Stoll et al., 1994; De León, 2001; Belli et al., 2012; Holder, 2017; Ho et al., 2021). VPA use during childbearing years has raised the most concern since clinical research has identified many risks associated with its use in pregnancy, highlighting birth defects and an increased risk of autism spectrum disorders (ASD) in offspring (Christensen et al., 2013; reviewed in Roullet et al., 2013). The absolute risk of ASD due to VPA usage in pregnancy was estimated as less than 5% (Christensen et al., 2013).

Impairments in social interaction and communication, as well as the presence of restricted and repetitive behaviors are the core symptoms of ASD (Farmer et al., 2013). ASD is also related to pain hyposensitivity/impaired cognitive processing of pain (Klintwall et al., 2011; Yasuda et al., 2016), increased anxiety and decreased interest in novelty (Vasa and Mazurek, 2015; Vivanti et al., 2018), and a higher risk for increased weight and obesity (Dhaliwal et al., 2019). The contribution of motor impairments to the pathophysiology of different neuropsychiatric conditions, including ASD (Bhat et al., 2011), is a growing field of research. In humans, movement is linked to exploration and is required for full perception of the world and receiving perceptual information from the environment (Bhat et al., 2011). It has been reported that children with ASD spend less time actively exploring their environment (Pierce and Courchesne, 2001). Also, recent findings suggest that in ASD subjects there is a decrease in attention to novel non-social stimuli, i.e., that reduced “novelty bias” might disproportionally affect learning in the social domain (Vivanti et al., 2018). In the first systematic review by Canu et al. (2021) indicated that non-social aspects of ASD become visible early during children’s development, before social impairments are manifested, and contribute to ASD via separate pathways. At a workshop convened by the Autism Science Foundation, it was highlighted that expanding investigation beyond the core domains of ASD should be encouraged (Silverman et al., 2022). The outcomes of intrauterine VPA exposure in ASD-undiagnosed children and adolescents are largely lacking. Focusing on non-core or associated symptoms of ASD, which also could have a strong impact on the quality of life of the general population (Oakley et al., 2021), could provide a more comprehensive picture of the outcomes of intrauterine VPA exposure. The assessment of the consequences of continuous intrauterine exposure to pharmacological agents is important and was recently highlighted by the finding that there is no safe threshold regarding alcohol consumption during pregnancy as children with even the lowest levels of exposure demonstrate poorer psychological and behavioral outcomes (including internalizing and externalizing psychopathology, attention deficits and impulsiveness) as they enter adolescence (Lees et al., 2020).

Adolescence is conserved across mammalian species, thus allowing the use of rodent models in the study of adolescent development (Schneider, 2013). However, there are still no published data dealing with the behavioral characteristics of adolescent offspring exposed to VPA during intrauterine development that would provide a satisfactory model for the continued use of VPA in pregnancy for the treatment of epilepsy as a chronic disorder (Borgelt et al., 2016; Błaszczyk et al., 2022). Behavioral alterations in the offspring of pregnant dams (mice, rats) exposed to different doses of VPA during the second and/or third weeks of pregnancy (Markram et al., 2007; Wlodarczyk et al., 2012) include impaired social interactions, increased repetitive behaviors, enhanced anxiety, locomotor hyperactivity, lower sensitivity to pain, higher sensitivity to non-painful sensory stimulation, impaired prepulse inhibition, enhanced eyeblink conditioning, impaired swim performance, and surface righting. Sex differences in the broad spectrum of behavioral, neuroendocrine and immunological aberrations in rats exposed to VPA on day 12.5 of gestation were described for the first time by the research group of prof. Przewłocki in a rodent model that exhibits several brain stem and cerebellar abnormalities resembling those found in the brain of autistic patients (Schneider et al., 2008). Male VPA rats exhibited lower sensitivity to pain, increased repetitive/stereotypic-like activity, higher anxiety, a decreased level of social interaction, an increased basal level of corticosterone, decreased weight of the thymus, decreased proliferative response of splenocytes to concanavalin A, a lower interferon-gamma/IL-10 ratio, and increased production of nitric oxide by peritoneal macrophages, while female VPA rats displayed only increased repetitive/stereotypic-like activity and a decreased interferon-gamma/IL-10 ratio (Schneider et al., 2008). The study confirmed the existence of similarities between the observed pattern of aberrations in VPA rats and features of disturbed behavior and immune function in autistic patients. The authors suggested that attenuation of behavioral, endocrine and immunological aberrations in VPA females might resemble disproportion in boys to girls ratio in autism, and emphasized the need for systematic investigation of sex differences in animal models of developmental disorders (Schneider et al., 2008).

The VPA-induced ASD-like animal model has been widely used to examine the neurobiology associated with affected responses to social context and social novelty (Hughes et al., 2020; Kuo and Liu, 2022). Findings obtained in the VPA model of ASD revealed communication deficits in VPA-exposed rats during infancy, adolescence and adulthood, with the most pronounced impact on adolescent animals (Gzielo et al., 2020). While the social play deficit in VPA-exposed adolescent rats was restricted to males, both males and females demonstrated a decreased number of ultrasonic vocalization calls (Gzielo et al., 2020). Yet, modifications of the responses to non-social stimuli and motor behavior in VPA-exposed offspring have gained much less attention (Al Sagheer et al., 2018; Quezada et al., 2021). In satiated rats, the exploration of a novel environment represents information-gathering behavior directed toward the reduction of environmental uncertainty and the possibility of finding an escape (Exner and Clark, 1993; Inglis et al., 2001). Monitoring the extent and the pattern of motor activity of VPA-exposed rodents in a novel environment can help to capture the potential relationship between behavioral and emotional problems related to the exposure. The interplay between the exploratory drive (approach motivation) and the avoidance response (avoidance motivation) is crucial for the overall behavioral responses to novelty (McGregor et al., 2020).

An important fact that should be emphasized here is that even though epilepsy requires continuous treatment during pregnancy, there is no animal model that examines the effects of continuous application of VPA during gestation. In that context, our previous study was a breakthrough as it focused for the first time on the early physical and motor postnatal development of offspring of mothers continuously exposed to gradually increasing doses of VPA (50–400 mg/kg/day) (Podgorac et al., 2016). It showed that delayed somatic development and postponed maturation of the motor system exist in all of the offspring prenatally exposed to VPA, with stronger effects observed at higher doses. Our findings imply that the strategy of continuous monitoring of general health and achievements in motor milestones in prenatally VPA-exposed offspring could help in recognizing early developmental irregularities and correcting them, thus providing an opportunity to facilitate the structural and functional maturation of the nervous system (Denckla, 2005; Bhat et al., 2011). Although it better models the therapeutic use of VPA in the human population, the model of continuous usage of the drug during pregnancy has not found wider assessment in preclinical studies.

The current study is an extension of our previous work (Podgorac et al., 2016). It examines the physical and psychomotor development of adolescent mice exposed to VPA during the entire intrauterine development, with an emphasis on dose-related effects within sexes. We have considered the adolescent phase in mice as follows: early [postnatal days (PND) 23–35], middle (PND36-48), late (PND49-61) adolescence (Adriani et al., 2004), with early adolescence as an important period for mouse growth (Holtrup et al., 2017) and spinal pain circuits maturation (Hathway et al., 2012), and middle adolescence as a period when mice exhibit behavioral characteristics that resemble those found in human adolescents (Adriani et al., 2004). Body weight (BW) measurement and the outcomes of the hot plate test (on PND25 and PND32), the elevated plus-maze test (on PND35), and the open field test (on PND40) were used to assess physical growth, the supraspinal reflex response to a painful thermal stimulus and conditional learning, anxiety-like/risk-assessment behavior, and novelty-induced psychomotor activity, respectively. The study did not aim to assess core autism traits but rather the associated mental health symptoms (sensorimotor function, anxiety, environmental novelty-driven psychomotor activity) and potential physical health concerns (BW and weight gain) as they could have a strong impact on the quality of life of the general population (Oakley et al., 2021).

## Materials and methods

### Animals

The study was performed on 8-week-old female NMRI mice that were housed under standard conditions (23 ± 2°C, 60–70% relative humidity and a 12-h light/dark cycle), and mated for the first time (2 females and 1 male per cage). Their offspring were tested at a specific PND, starting from PND25 to PND40, thus covering early and middle adolescence in mice (Adriani et al., 2004).

All animal procedures were in compliance with Directive 2010/63/EU on the protection of animals used for experimental and other scientific purposes, and were approved by the Ethical Committee for the Use of Laboratory Animals of the Institute for Biological Research, University of Belgrade (04-05/15).

### Experimental procedure

According to the treatment, 5 experimental groups were formed (7 females per group), with one control group treated with saline (CON) and 4 groups treated with VPA at doses of 50 mg/kg (VPA-50), 100 mg/kg (VPA-100), 200 mg/kg (VPA-200), and 400 mg/kg (VPA-400). These doses correspond to human daily doses of 4, 8, 16, and 32 mg/kg/day or 240, 480, 960, and 1,920 mg/day, respectively. Dose translation, which provides the conversion of a dose for animals into a dose for humans and after reversion the conversion of a dose for humans into a dose for animals, is based on the body surface area and was made using the US FDA guideline [ U.S. Food and Drug Administration [FDA], 2005; for more details refer to Podgorac et al. (2016)].

The saline/VPA treatment was performed every morning between 08:00 and 09:00 a.m. from the moment of mating and throughout the entire period of breeding and gestation. Females were treated subcutaneously (s.c.) into the loose skin on the back of the neck with an appropriate dose of the sodium salt of VPA (P4543, Sigma-Aldrich, MO, United States) dissolved in saline to a final volume of 2.5 ml/kg or the corresponding volume of saline.

After the birth of offspring, the treatment was stopped and the mothers with their offspring were moved to separate cages. The offspring remained with the mothers until PND21, after which they were separated by sex and subjected to assessment of the BW and state of the motor and sensory system at the specific stage of postnatal development. One male and one female per litter were randomly chosen for BW measurement and behavioral monitoring, starting from PND25, to avoid the litter effect (Lazic and Essioux, 2013; Jiménez and Zylka, 2021). Thus, the number of animals of each sex per group was *n* = 7 and the litter was the statistical unit of analysis. All offspring were without visible signs of physical malformations.

A battery of behavioral tests, including the hot plate test (HPT), the elevated plus-maze test (EPMT) and the open field test (OFT), was used to examine the supraspinal reflex response to a painful thermal stimulus and conditional learning, anxiety-like/risk-assessment behavior and novelty-induced psychomotor activity, respectively.

### Monitoring of the body weight

Body weight was monitored as an objective physiological and welfare indicator (Hawkins et al., 2011). The animals were weighed using an electronic balance on PND25 and PND32 considering that PND18-34 is an important period for growth in mice (Holtrup et al., 2017).

Measurement was performed 1 h before behavioral testing. Each animal was confined in a perforated chamber that was tared before enclosing the rat. Weight gain was calculated as follows: Delta (Δ) BW = BW on PND32 (g) – BW on PND25 (g).

### Behavioral apparatus and testing

Behavioral testing was performed in a room with controlled lighting, temperature and noise from 9 a.m. to 2 p.m. Mice were transported to the room at least 1 h before testing. The equipment was cleaned with 20% ethanol to eliminate any scent traces from the previous animal.

#### Hot plate test

The HPT is the most widely used experimental method to assess nociception in rats and mice and if repeated during a critical period, it can serve as a model for conditional learning (Le Bars et al., 2001). The advantages of this test are that it is objective and quantifiable, that it can be administered repeatedly without causing inflammation, and that it assesses supraspinally organized responses to a noxious stimulus (Gunn et al., 2011).

The HPT was performed on PND25 and repeated on PND32. The mouse was placed on a 50 ± 1°C hot plate and the latency of lifting and shaking the hind paw was measured. The mouse was removed from the hot plate immediately when this response was observed or if no response occurred within 40 s. Although mice are typically tested on a significantly warmer hot plate [55°C, discussed in Gunn et al. (2011)], the conditions of the test (temperature and cut-off time) in the present study were set to avoid unnecessary nociceptive stimulation and tissue damage in the tested animals, since the lower hot-plate temperatures might be more appropriate if tested mice have the low threshold for thermal nociceptive stimuli (Vermeirsch and Meert, 2004). In previous studies, which used adult NMRI mice (that weighed 30 g on an average) for the HPT temperature of 50 ± 0.5°C the cut-off time was set to 30 s (Vermeirsch and Meert, 2004). Given that the mice used in this study were younger and had significantly less weight, the cut-off time was set to 40 s. The protocols of previous studies that used this cut-off time (even with a slightly higher plate temperature, i.e., 52°C) for mice weighing around 16 g (Tegeder et al., 2004) were considered.

A water bath was used to heat the plate (a metal cylinder immersed in water). Only those animals that met the criteria set by the test on PND25 were tested on PND32 as well. The difference in response latency between the two time points of interest was calculated as follows: Delta (Δ) response latency = latency on PND25 (s) − latency on PND32 (s).

#### Elevated plus-maze test

The EPMT is a widely used behavioral test for the assessment of anxiety-like behavior in rodents with an increase in open arm activity (entries and/or duration) reflecting anti-anxiety behavior (Walf and Frye, 2007). Other ethological measures, which can be considered as “risk assessment,” are the number of rears, head dips and stretched-attend postures (Rodgers et al., 1999; Albrechet-Souza et al., 2007; Walf and Frye, 2007).

The EPMT was conducted on PND35 and each mouse was tested only once. The apparatus consisted of two open arms (30 cm × 5 cm) and two closed arms (30 cm × 5 cm × 15 cm) extending from a central open square (5 cm × 5 cm), elevated on a pedestal to a height of 45 cm above the floor. The floor of the maze was constructed from gray Plexiglas and the side walls were made of clear Plexiglas. A gray Plexiglas lip (5 mm high) was attached to the floor of the open arms to prevent the mice from falling off. The mice were placed individually in the central open square, facing one of the open arms, and were recorded for 5 min.

Quantitative parameters such as the number of entries and the time spent in open and closed arms, as well as the anxiety index were analyzed to assess anxiety-like behavior. The anxiety index was calculated according to Cohen et al. (2013) as follows: Anxiety Index = 1 − [([Open arm time/Test duration] + [Open arms entries/Total number of entries])/2]. Entry was defined as the placement of all four paws into an arm.

The qualitative parameters such as the number of rears (standing on hind legs), unprotected head dipping (lowering the head over the sides of the open arms toward the floor), and protected stretched-attend postures (forward elongation of the head and shoulders followed by retraction to the original position, from the protected areas of the maze) were analyzed to evaluate risk-assessment behavior.

#### Open field test

Spontaneous activity of animals in the open rectangular arena was used to assess locomotor, stereotypy-like and rearing activity of the animals in response to a novel inescapable environment. In rodents, motor (particularly locomotor) activity in a novel environment models sensation seeking and is used in preclinical studies to examine the role of behavioral/personality traits in the acquisition of drug-taking behavior (Dellu et al., 1996).

The OFT was conducted on PND40 using an automatic device, the Auto-Track System (Version 3.0A, Columbus Inc., OH, United States). The mouse was placed in the arena and allowed to explore it freely for a single 30-min session. Monitoring cages (Opto-Varimex) are constructed of Plexiglas, dimensions 44.2 cm × 43.2 cm × 20 cm, and are connected to the Auto-Track interface. Each cage is equipped with one grid of infrared beams placed around the arena and the other one placed a few centimeters above the floor. By interrupting the beams, an analog signal is generated and sent to a computer (connected to the Auto-Track interface) where digital signal conversion is performed, i.e., certain activities are counted. The system can record 11 behavioral parameters, including locomotor (horizontal exploratory) activity, stereotypy-like activity and rearing (vertical exploration). The type of activity is characterized by the mouse’s movements and is determined by a user-defined box size (in this experiment set to two beams). Thus, locomotor activity is defined as the interruption of two consecutive beams, stereotypy-like activity as a movement within the space defined by two intersected beams, and rearing as the interruption of beams placed above the floor.

### Statistical analysis

A software package STATISTICS 6.0 (StatSoft Inc., Tulsa, OK, United States) was used for statistical analysis of the obtained results. Although the experimental design suggested that the most appropriate statistical analysis of the results would be two-way ANOVA with the VPA treatment (dose) and sex of the examined offspring as factors, we could not perform this analysis because several data sets did not have a normal distribution even after data transformation. The normality of the data sets was estimated by Shapiro–Wilk’s test (Razali and Wah, 2011). Therefore, statistical comparisons between groups of the same sex were performed by Kruskal–Wallis ANOVA [using the dose of VPA as a factor, i.e., 0 (control), 50, 100, 200, and 400 mg/kg/day] and, when applicable, it was followed by a *post hoc* Mann–Whitney *U*-test. Differences were considered significant if *p* < 0.05.

## Results

### Body weight

Intrauterine exposure to VPA significantly affected the BW of females and males on PND25 [*H*_(4,35)_ = 13.605, *p* = 0.009 and *H*_(4,35)_ = 12.714, *p* = 0.013, respectively] and PND32 [*H*_(4,35)_ = 17.789, *p* = 0.001 and *H*_(4,35)_ = 10.774, *p* = 0.029, respectively].

**FIGURE 1:**
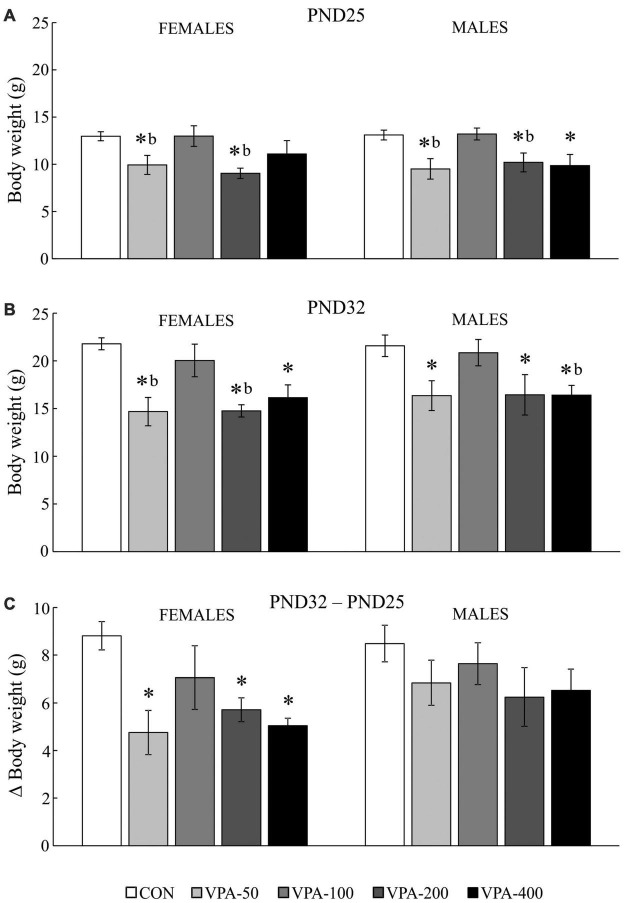
Average body weight (BW) of adolescent female and male mice prenatally exposed to VPA at doses of 50 mg/kg (VPA-50), 100 mg/kg (VPA-100), 200 mg/kg (VPA-200), and 400 mg/kg (VPA-400). The animals were weighed on PND25 **(A)** and PND32 **(B)**; based on the obtained parameters weight gain was calculated **(C)** as the BW on PND32 (g) – BW on PND25 (g). Each bar represents the mean ± SEM (*n* = 7 mice/group/sex). *p < 0.05 compared to the control (CON) group treated with saline; *^b^p* < 0.05 compared to the VPA-100 group (*U*-test).

On PND25, the VPA-50 and VPA-200 groups of offspring of both sexes as well as the VPA-400 group in males had a significantly reduced BW compared to the sex-matched CON groups (Figure 1A; *p < 0.05, *U*-test). The VPA-50 and VPA-200 groups weighed less than the VPA-100 group in both females and males (Figure 1A; *^b^p* < 0.05, *U*-test).

**FIGURE 2:**
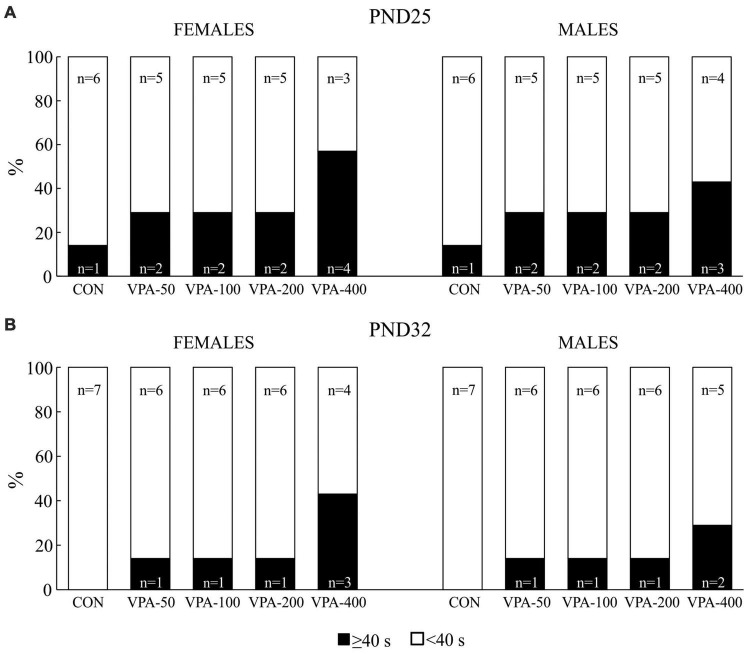
Percentage of the total number of adolescent female and male mice prenatally exposed to VPA at doses of 50 mg/kg (VPA-50), 100 mg/kg (VPA-100), 200 mg/kg (VPA-200), and 400 mg/kg (VPA-400) that showed latencies less than 40 s (<40 s) and equal to or higher than 40 s (≥40 s) on PND25 **(A)** and PND32 **(B)** in the hot plate test. The control (CON) group was treated with saline.

On PND32, all VPA-exposed groups except VPA-100 had significantly lower BW compared to the matching CON group in both females and males (Figure 1B; *p < 0.05, *U*-test). In females, the VPA-50 and VPA-200 groups weighed less than the VPA-100 group (Figure 2B; *^b^p* < 0.05, *U*-test). In males, the VPA-400 group weighed less than the VPA-100 group (Figure 1B; *^b^p* < 0.05, *U*-test).

There were no significant differences in the BW of females and males on PND25 and PND32. All experimental groups of animals increased their BW from PND25 to PND32. Importantly, the analysis of weight gain showed that in females there was a significant difference in weight gain between groups [*H*_(4, 35)_ = 11.388, *p* = 0.023]; namely, it was significantly lower in all VPA groups, except in VPA-100 compared to the CON group (Figure 1C; *p < 0.05, *U*-test), while in males there were no significant differences in weight gain between groups [*H*_(4, 35)_ = 4.717, *p* = 0.318].

### Hot plate test

**FIGURE 3:**
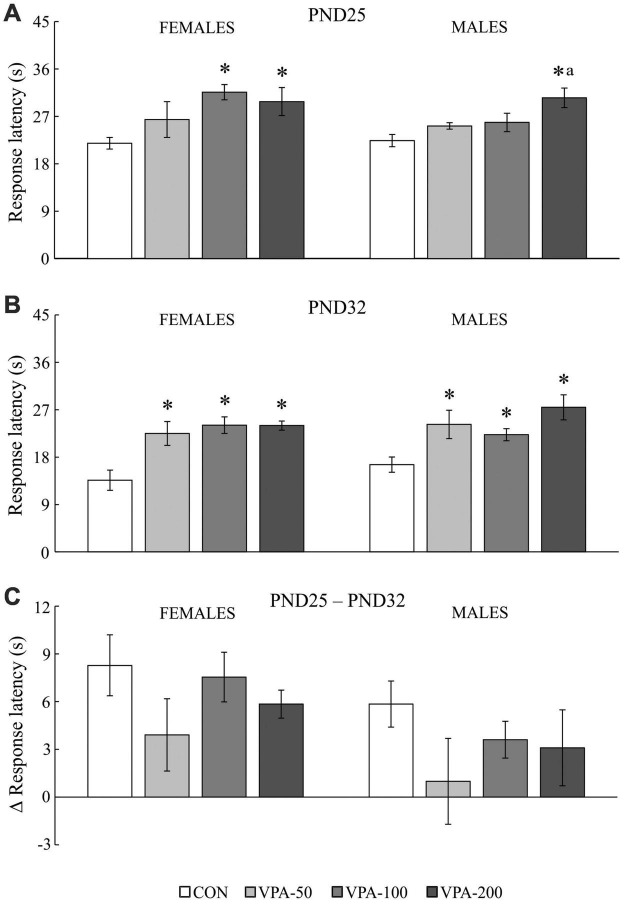
The response latency to the thermal stimulus in adolescent female and male mice prenatally exposed to VPA at doses of 50 mg/kg (VPA-50), 100 mg/kg (VPA-100), and 200 mg/kg (VPA-200), which showed latencies less than 40 s in the hot plate test. The animals were tested on PND25 **(A)** and PND32 **(B)**; based on the obtained parameters, the difference in response latency between two time points of interest was calculated **(C)** as the latency on PND25 (s) – latency on PND32 (s). Females and males in the VPA-400 group were not considered because a small number of them showed latencies less than 40 s. Each bar represents the mean ± SEM (CON group: *n* = 6 mice/sex; VPA groups: *n* = 5 mice/group/sex). *p < 0.05 compared to the control (CON) group treated with saline; *^a^p* < 0.05 compared to the VPA-50 group (*U*-test).

Two types of data were obtained in the HPT, qualitative data obtained by assessing the number of animals per group that showed latencies of less than 40 s (Figure 2), and quantitative data obtained by analyzing the responses of these animals in more detail (Figure 3). Those animals showing latencies equal to or higher than 40 s were discarded.

On PND25, all experimental groups of mice contained animals that showed latencies equal to or higher than 40 s in the HPT, although their number was higher in the VPA-exposed than in the CON group, particularly considering the VPA-400 group in both sexes (Figure 2A). When the animals were tested on PND32, the number of females and males that showed latencies equal to or higher than 40 s was lower in all VPA groups, while it was completely absent in the CON group (Figure 2B). Quantitative response analysis for the VPA-400 group was not performed because in both sexes, only about half of the animals showed latencies lower than 40 s.

The analysis of the response latency to the thermal stimulus in mice that showed latencies less than 40 s (except for the VPA-400 group; Figure 3) showed a significant influence of VPA dose on the examined parameter in both sexes and in both examined time points of interest, i.e., PND25 [females: *H*_(3, 21)_ = 9.212, *p* = 0.027; males: *H*_(3, 21)_ = 9.160, *p* = 0.027] and PND32 [females: *H*_(3, 21)_ = 12.485, *p* = 0.006; males: *H*_(3, 21)_ = 11.217, *p* = 0.011].

On PND25, a significant increase in the response latency was observed in females of the VPA-100 and VPA-200 groups and in males of the VPA-200 group, compared to sex-matched CON groups (Figure 3A; *p < 0.05, *U*-test). On PND32, a significant increase in the response latency was observed in all VPA-exposed groups in both sexes when compared to the CON groups (Figure 3B; *p < 0.05, *U*-test). There were between-group differences only in males between the VPA-50 and VPA-200 groups on PND25 (Figure 3A; *^a^p* < 0.05 vs. VPA-50, *U*-test).

The values of the response latency in the HPT were generally lower on PND32 than on PND25 in animals of both sexes, and a detailed analysis of the delta (Δ) response latency showed that the dose of VPA did not have a significant influence on this parameter in animals of either sex [Figure 3C; females: *H*_(3, 21)_ = 2.517, *p* = 0.472; males: *H*_(3, 21)_ = 2.502, *p* = 0.475]. As noted above, females and males in the VPA-400 group were not considered for this analysis because only a small number of them showed latencies lower than 40 s in the HPT.

### Elevated plus-maze test

The VPA dose as a factor showed significant influence on the behavior of exposed mice in the EPMT on PND35 (Table 1), inducing significant changes in the following behavioral parameters: the number of entries in the open arms [females: *H*_(4, 35)_ = 20.555, *p* = 0.001; males: *H*_(4, 35)_ = 15.401, *p* = 0.004], the time spent in the open arms [females: *H*_(4, 35)_ = 10.784, *p* = 0.029; males: *H*_(4, 35)_ = 14.372, *p* = 0.006], the time spent in the closed arms [females: *H*_(4, 35)_ = 11.303, *p* = 0.023; males: *H*_(4, 35)_ = 12.232, *p* = 0.016], the anxiety index [females: *H*_(4, 35)_ = 14.147, *p* = 0.007; males: *H*_(4, 35)_ = 18.158, *p* = 0.001], the number of head dips [females: *H*_(4, 35)_ = 15.222, *p* = 0.004; males: *H*_(4, 35)_ = 12.110, *p* = 0.017] and stretched-attend postures [females: *H*_(4, 35)_ = 20.999, *p* = 0.001; males: *H*_(4, 35)_ = 10.882, *p* = 0.028].

**TABLE 1:** Quantitative and qualitative parameters of behavior in EPMT of adolescent female and male mice prenatally exposed to VPA at doses of 50 mg/kg (VPA-50), 100 mg/kg (VPA-100), 200 mg/kg (VPA-200), and 400 mg/kg (VPA-400) on the PND35.

		Number of entries		Time		Anxiety index	Rears	Head dips	Stretched-attend postures
			
		Open arms	Closed arms	Open arms	Closed arms				
FEMALES	CON	5 ± 1	11 ± 1	57 ± 9	157 ± 16	0.75 ± 0.04	30 ± 4	21 ± 2	89 ± 10
	VPA-50	9 ± 1*	8 ± 1	115 ± 16*	91 ± 17*	0.54 ± 0.04*	24 ± 3	34 ± 5*	82 ± 4
	VPA-100	3 ± 1^a^	10 ± 1	37 ± 15^a^	160 ± 18^a^	0.84 ± 0.03^a^	26 ± 3	16 ± 2^a^	63 ± 4^a^
	VPA-200	4 ± 0^a^	9 ± 1	50 ± 13^a^	167 ± 10^a^	0.76 ± 0.04^a^	20 ± 5	17 ± 3^a^	82 ± 4^b^
	VPA-400	3 ± 1^a^	8 ± 1	59 ± 15	153 ± 11^a^	0.76 ± 0.06^a^	22 ± 3	15 ± 2^a^	45 ± 3* ^abc^
MALES	CON	6 ± 1	9 ± 2	81 ± 19	123 ± 21	0.65 ± 0.07	26 ± 4	30 ± 4	67 ± 6
	VPA-50	8 ± 1	9 ± 1	90 ± 19	112 ± 14	0.63 ± 0.05	25 ± 3	29 ± 5	89 ± 5*
	VPA-100	3 ± 0* ^a^	11 ± 1	24 ± 4* ^a^	187 ± 16* ^a^	0.87 ± 0.01* ^a^	30 ± 3	20 ± 3	62 ± 5^a^
	VPA-200	5 ± 2	8 ± 1^b^	67 ± 20	145 ± 26	0.71 ± 0.06^b^	19 ± 7	22 ± 7	72 ± 7
	VPA-400	3 ± 0* ^a^	12 ± 2^c^	24 ± 9*^a^	181 ± 12* ^a^	0.87 ± 0.02* ^ac^	21 ± 4	11 ± 2* ^ab^	57 ± 7^a^

Each value represents the mean ± SEM (*n* = 7 mice/group/sex). *p < 0.05 compared to the control (CON) group treated with saline; ^*a*^*p* < 0.05 compared to the VPA-50 group; ^*b*^*p* < 0.05 compared to the VPA-100 group; ^*c*^*p* < 0.05 compared to the VPA-200 group (*U*-test). The bold values indicate significant changes.

In females, a significantly higher number of entries and longer times spent in the open arms, a lower anxiety index, shorter times spent in the closed arms and a higher number of head dips were detected in the VPA-50 group compared to the CON group (Table 1; *p < 0.05, *U*-test). In the VPA-100 and VPA-200 groups, all examined parameters did not differ compared from the CON group (Table 1). Importantly, the only parameter that differed in the VPA-400 group from the CON group was a lower number of stretched-attend postures (Table 1; *p < 0.05, *U*-test), and this parameter was also lower than that observed in VPA-50, VPA-100, and VPA-200 groups (Table 1; a, b, and c, respectively, *p* < 0.05, *U*-test).

In males a significantly higher number of stretched-attend postures in the VPA-50 group was the only parameter that differed in the behavior of VPA-50 males in the EPMT from the CON males (Table 1; *p < 0.05, *U*-test). The VPA-100 and VPA-400 groups differed from the CON group regarding the anxiety index (which was increased in the VPA-exposed animals), and parameters based on which the anxiety index was calculated (Table 1; *p < 0.05, *U*-test). The VPA-400 group also had a significantly lower number of head dips than the CON group (Table 1; *p < 0.05, *U*-test). The lower number of head dips in the VPA-400 than in the VPA-100 group was the only significant difference in the behavior of these animals in the EPMT. Detailed analysis of the differences between VPA groups regarding all 8 parameters analyzed to assess the behavior of the animals in the EPMT is given in Table 1.

The VPA-200 group of males did not differ from the CON group regarding all analyzed parameters of EPMT-related behavior. Since the same was observed in the group of VPA-200 females, we concluded that based on the current findings, prenatal exposure to VPA at a dose of 200 mg/kg/day does not significantly influence the behavior of the offspring of both sexes in the EPMT.

### Open field test

**FIGURE 4:**
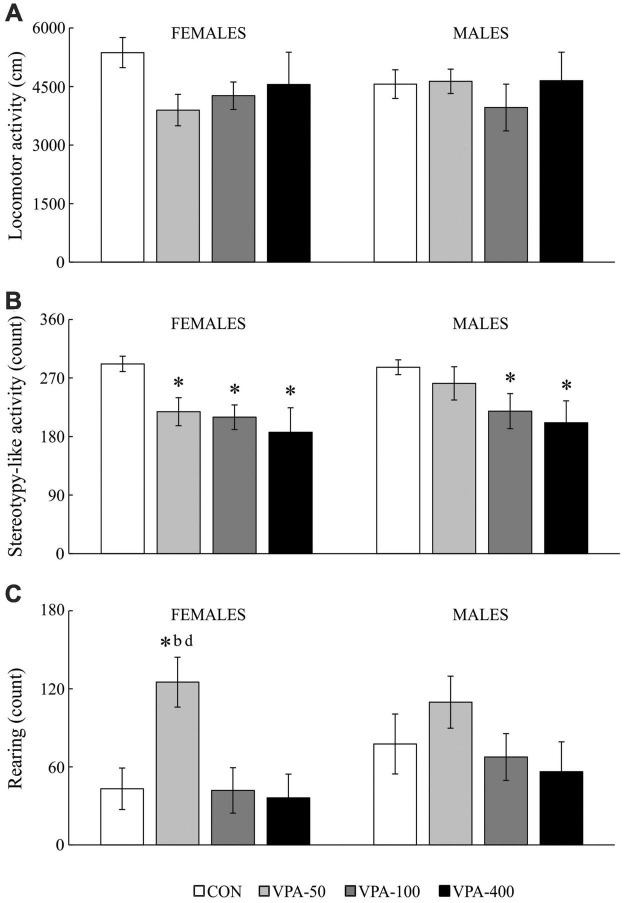
Locomotor activity **(A)**, stereotypy-like activity **(B)**, rearing **(C)** in adolescent female and male mice prenatally exposed to VPA at doses of 50 mg/kg (VPA-50), 100 mg/kg (VPA-100), and 400 mg/kg (VPA-400) on the PND40. The VPA-200 group was not examined in the OFT because of the absence of behavioral changes in the EPMT. Each bar represents the mean ± SEM (*n* = 7 mice/group/sex). *p < 0.05 compared to the control (CON) group treated with saline; *^b^p* < 0.05 compared to the VPA-100 group; *^d^p* < 0.05 compared to the VPA-400 group (*U*-test).

Open field test testing was performed to check whether the behavior of the animals in the EPMT is related to changes in general motor activity (note that the VPA-200 group was not examined in the OFT due to the absence of behavioral changes in the EPMT). The data obtained in the tested animals (Figure 4) showed that locomotor activity was not changed in the VPA-exposed animals of both sexes (Figure 4A). The other two examined parameters (stereotypy-like activity and rearing) showed dose-related changes within sexes.

In females, VPA-exposure was related to affected stereotypy-like activity [Figure 4B; *H*_(3, 28)_ = 10.427, *p* = 0.015] and rearing activity [Figure 4C; females: *H*_(3, 28)_ = 9.536, *p* = 0.023]. The *post hoc* test revealed a significant decrease in stereotypy-like activity in all VPA groups compared to the CON group (Figure 4B; *p < 0.05, *U*-test) while rearing activity was increased in the VPA-50 group compared to the CON group (Figure 4C; *p < 0.05, *U*-test), VPA-100 group (Figure 4C; *^b^p* < 0.05, *U*-test) and VPA-400 group (Figure 4C; *^d^p* < 0.05, *U*-test).

In males, VPA exposure was associated only with changes in stereotypy-like activity [Figure 4B; *H*_(3, 28)_ = 8.976, *p* = 0.030]. The *post hoc* analysis revealed a significant decrease in this parameter in the VPA-100 and VPA-400 groups compared to the control group (Figure 4B; *p < 0.05, *U*-test).

### Summary of the obtained results

To have a better insight into the results obtained in the examined model, a summarized tabular presentation of the results is provided in Table 2. It is clear that in both female and male offspring no dose does not change at least one of the analyzed parameters.

**TABLE 2:** Summary of physical, nociceptive, emotional, and psychomotor responses of adolescent female and male mice exposed to VPA at doses of 50 mg/kg (VPA-50), 100 mg/kg (VPA-100), 200 mg/kg (VPA-200), and 400 mg/kg (VPA-400).

	Females				Males			
		
	VPA-50	VPA-100	VPA-200	VPA-400	VPA-50	VPA-100	VPA-200	VPA-400
**Physical response**								
Body weight on PND25	↓	/	↓	/	↓	/	↓	↓
Body weight on PND32	↓	/	↓	↓	↓	/	↓	↓
Δ Body weight	↓	/	↓	↓	/	/	/	/
**Nociception**								
Response latency on PND25	/	↑	↑	N/A	/	/	↑	N/A
Response latency on PND32	↑	↑	↑	N/A	↑	↑	↑	N/A
Δ Response latency	/	/	/	N/A	/	/	/	N/A
**Emotional response**								
Anxiety index	↓	/	/	/	/	↑	/	↑
**Risk-assessment behavior**								
*Unprotected head dipping*	↑	/	/	/	/	/	/	↓
*Protected stretched-attended postures*	/	/	/	↓	↑	/	/	/
**Psychomotor response**								
Locomotor activity	/	/	N/A	/	/	/	N/A	/
Stereotypy-like activity	↓	↓	N/A	↓	/	↓	N/A	↓
Rearing	↑	/	N/A	/	/	/	N/A	/

Increase (↑), decrease (↓), no effect (/), and not applicable (N/A).

## Discussion

Prenatal exposure to VPA could have long-term effects even in exposed individuals that have not been diagnosed with birth defects and ASD; however, an experimental approach directed toward the elucidation of the dose-related long-term outcomes within sexes is missing. This study shows in a mouse model that continuous intrauterine exposure to VPA results in a dose-related decrease in BW in adolescent animals of both sexes along with dose-specific behavioral outcomes within sexes in terms of the supraspinal reflex response to a painful thermal stimulus (without affecting the reduction of the response latency to the thermal stimulus due to repeated testing), anxiety-like and risk-assessment behavior, and novelty-induced psychomotor activity. The study highlights that BW and behavioral consequences of continuous intrauterine exposure to VPA are delicate for assessment in adolescent offspring since they are dose-related within sexes. As the doses of VPA used in the mouse model were calculated to cover both the subtherapeutic and therapeutic dose range of VPA, the findings suggest that an experimental approach based on a narrow range of therapeutic doses of VPA should be avoided because of the incomplete picture of the safety of VPA usage during pregnancy. Moreover, it draws attention to the influence of low (subtherapeutic) doses of VPA on the BW and the psychomotor characteristics of the adolescent offspring, thus providing important guidelines for future research and emphasizing that there should be no equation between the terms “low dose” and “safe dose” regarding the use of VPA in pregnancy.

### The influence of continuous prenatal exposure to valproic acid on the body weight of offspring

The model of continuous maternal exposure to increasing doses of VPA (50–400 mg/kg/day), which roughly correlate to those used in clinical practice (200–3,600 mg/day; Roullet et al., 2013) was used for the first time in our previous study (Podgorac et al., 2016). We observed a fine dose-related impact of prenatal treatment with VPA on postnatal BW gain, highlighting that on PND15 all tested doses of VPA, except the dose of 100 mg/kg/day, exhibit uniform negative effects on BW. The current study extends this knowledge by showing that the same dose-related negative influence of prenatal exposure to VPA on the BW of the offspring, regardless of sex, is also evident during the period of adolescent growth in mice (i.e., on PND25 and PND32; Holtrup et al., 2017), which belongs to the early adolescent developmental period in rodents (Adriani et al., 2004). It appears that the impact of prenatal VPA exposure on the BW of the offspring is more consistent and easier to define than the impact on behavior (discussed below); however, changes in BW should not be considered as a physiological biomarker of the psychomotor/emotional consequences of the exposure, at least not in the model used here. The findings on the changes in BW of prenatally VPA-exposed offspring are not consistent; thus for example, the study performed on the offspring of dams treated with VPA at a dose of 600 mg/kg on the 13th day of gestation reported significantly higher BW in exposed as compared to control animals from PND18 to PND22 females, and PND17 males (Wagner et al., 2006). This disagreement in the observed effects of VPA could be attributed to the animal model used because there are clear indications that some consequences of VPA administration are directly correlated with the metabolism of the substance in the mother and the formation of active metabolites, which depends on the route of administration, treatment duration and the dose of VPA (Lloyd, 2013). A lower BW throughout postnatal development in VPA-exposed offspring was reported in a study performed using socially monogamous prairie voles (Sailer et al., 2019). The authors concluded that the observed effect is probably related to neurodevelopmental alterations, since weight gain in VPA-exposed offspring was control-like. In the present study, the gain in BW was unchanged only in male offspring, while in VPA-exposed female offspring it was lower in all groups that exhibited lower BW as compared to the control. These findings highlight that the reasons for the lower BW in prenatally VPA-exposed adolescent mouse offspring are sex-specific, and in males they most likely have neurodevelopmental causes, while in females they may be also related to effects on the metabolic processes specific to this period. From the methodological point of view, these findings emphasize that the metric for BW (change or raw value) should be taken into account for between-study comparison, particularly when considering sex differences. Mice show increased food and energy expenditure during adolescence (Schneider et al., 2000; Pann et al., 2020); however, examination of the metabolic and endocrine signature of prenatal VPA exposure is outside the scope of this study, so no data on this topic are available for the model. Overall, this part of the study conducted using the mouse model showed that only prenatal exposure to VPA at a dose of 100 mg/kg/day does not affect the BW of the adolescent offspring of both sexes. This dose corresponds to a daily human dose of about 500 mg/day. Table 2 shows how this part of the results stands for the other examined parameters.

### The influence of continuous prenatal exposure to valproic acid on offspring behavior in the hot plate test

The results presented herein revealed that prenatal exposure to VPA in a wide dose range is related to the modified response to a painful thermal stimulus during adolescence, i.e., hyposensitivity to thermal pain. All tested doses elicited an effect on PND32 in animals of both sexes, while in PND25 the influence of the lower doses is not evident in males, suggesting that sex-related outcomes could be expected in younger adolescent VPA-exposed mice. The measurable outcomes of only higher VPA doses on the nociceptive response of the offspring on PND25 may reflect their stronger influence on the maturation of the pain-control system. Namely, it has been shown that in rodents the first three postnatal weeks are crucial for the formation of the nociceptive withdrawal reflex (Holmberg and Schouenborg, 1996), while postnatal week 4 is a critical period during which spinal pain circuits mature (Hathway et al., 2012). Nevertheless, up to PND32 the influence of all tested doses was evident. The latency of the response to the thermal stimulus in females and males of the control and all VPA-exposed groups was lower during repeated testing (on PND32) than on PND25, suggesting that memory of the previous experience might exist, which remains to be examined in detail in a study that will include a group of animals tested for the first time on PND32. Since the delta (Δ) response latency was similar in all experimental groups, this suggests that prenatal VPA exposure *per se* did not significantly contribute to the reduction of the response latency due to repeated testing of offspring in the HPT.

Studies that examined the VPA rodent model of autism also showed that VPA-exposed offspring exhibit increased latency to withdrawal from a thermal stimulus (Schneider and Przewłocki, 2005; Dendrinos et al., 2011).

### The influence of continuous prenatal exposure to valproic acid on offspring behavior in the elevated plus-maze test

The results of the present study revealed that prenatal exposure to VPA influences anxiety-like and risk-assessment behavior of the offspring in the EPMT in a dose-specific manner within sexes. In female offspring, only the highest and lowest tested doses of VPA possessed a behavioral signature: exposure to 400 mg/kg/day was related to a decrease in the number of protected stretched-attended postures, suggesting a decrease in risk-assessment/anxiety-like behavior in the given paradigm (Walf and Frye, 2007), while exposure to 50 mg/kg/day decreased the anxiety index and increased the number of unprotected head dips as an ethological measure of decreased anxiety (Rodgers and Johnson, 1995). In male offspring, the VPA-50 group showed an increased number of stretched-attended postures, which should be viewed as an increase in anxiety-like behavior, while males belonging to the VPA-100 and VPA-400 groups showed an increase in the anxiety index and a decrease in unprotected head dipping (only in the VPA-400 group). Importantly, both VPA-100 and VPA-400 males showed a highly comparable response in the EPMT (except for head dipping), suggesting that the dose-response relationship is weak. The number of entries into closed arms, which is used as an index of general activity (Rodgers and Dalvi, 1997), did not change either in females or in males, suggesting that prenatal exposure to VPA does not produce hyperactivity in the offspring to a novel environment. Overall, these findings revealed that: (i) prenatal exposure to VPA in female offspring is generally related to a decrease in anxiety-like behavior while in male offspring it is explicitly associated with an increase in anxiety, as assessed through both classical and ethological behavioral parameters in the EPMT paradigm; (ii) there is an absence of a dose-response relationship between the exposure dose and the adverse behavioral/emotional response of the offspring in the EPMT paradigm. This part of the study showed that only prenatal exposure to 200 mg/kg/day of VPA does not affect the behavior of the adolescent offspring of either sex in the EPMT; this dose corresponds to a daily human dose of 1,000 mg/day.

Studies performed on the VPA rodent model of ASD also showed elevated anxiety only in male animals (along with a lower sensitivity to pain, increased repetitive/stereotypic-like activity and a decreased number of social behaviors compared to control males), while the only behavioral alteration observed in female offspring was an increased number of and longer duration of repetitive/stereotypic-like movements (Schneider et al., 2008).

### The influence of continuous prenatal exposure to valproic acid on offspring behavior in the open field test

To additionally check whether changes in anxiety-related behavior of the VPA-exposed offspring in the EPMT are related to changes in general motor activity, the OFT was performed by exposing the animals to a novel rectangular arena. Of all tested VPA-exposed groups (VPA-50, VPA-100, and VPA-400, both females and males), the only observed increase was detected regarding vertical (rearing) activity in female mice belonging to the VPA-50 group. Another observed change was decreased stereotypy-like activity in all tested animals except males in the VPA-50. These results indicate that in the mouse model, exposure to VPA throughout the entire intrauterine development is not associated with the appearance of general hyperactivity in adolescence. Since increased locomotor activity in a novel environment in rodents models sensation-seeking traits in humans (Dellu et al., 1996), present findings indicate that there is no direct relationship between prenatal exposure to VPA and a sensation-seeking trait in adolescence.

Importantly, increased locomotor and repetitive, stereotypic-like hyperactivity combined with lower exploratory (rearing) activity was described in adolescent rats exposed to VPA on the 12.5th day of gestation (Schneider and Przewłocki. 2005) and in autistic patients (Schneider and Przewłocki, 2005). Locomotor activity in the open field is thought to be the result of competition between general arousal and exploratory activities, so increased non-goal-directed locomotor velocity may suppress exploration (Schneider and Przewłocki, 2005). Considering this and later findings (Wagner et al., 2006; Schneider et al., 2008; Zhang et al., 2012), it was concluded that prenatal VPA exposure induces varying degrees and types of repetitive behaviors in animal models (Mabunga et al., 2015) although, given the results of our study, this seems to be a generalized conclusion without much emphasis on the specificity of the animal model.

As we discussed previously (Pesić et al., 2010), locomotor activity and stereotypic behaviors are competing motor activities mediated by distinct mechanisms. The dopaminergic mesolimbic system has been directly implicated in the control of locomotor behavior, while the integrity of the dopaminergic input to the striatum (nigrostriatal system) probably plays a fundamental role in the control of stereotypic activity (Pijnenburg et al., 1976; Canales and Graybiel, 2000). Different components of stereotypic behavior could differ in their neuroanatomic substrates (Costall and Naylor, 1977). However, the motor stereotypy recorded in our study included all small movements, without differentiation between different forms of stereotypy. Due to the automatized measurement of motor activity, stereotypy is defined as any activity that is small in size and repetitive in nature and therefore these movements have been defined as stereotypy-like movements. The same apparatus for motor activity monitoring (and separation of certain activities) was used in a study dedicated to the VPA model of ASD (Schneider and Przewłocki, 2005).

The dissociation of locomotor and vertical activity in response to a novel environment as described herein is not a common phenomenon in behavioral studies but is thought to be associated with GABA_B_ receptor stimulation in areas of the brain that mediate reward (Zhou et al., 2005). An increase in vertical activity without a change in locomotor (horizontal) activity in the VPA-50 group of females could be a consequence of selective activation of GABA_B_ receptors in the motive circuit due to the rewarding effects of novelty (Wittmann et al., 2007). These data provide a good basis for further research on the impact of prenatal exposure to low doses of VPA on rewarding pathways and approach/avoidance motivation conflict in response to novelty (Aupperle and Paulus, 2010; McGregor et al., 2020).

### The importance of the rodent model of continuous exposure to valproic acid throughout the entire intrauterine development

The VPA animal model of ASD is one of the most widely used animal models in the field (Mabunga et al., 2015). Nevertheless, regardless of the wide usage of the VPA rodent model of ASD in many high-quality studies, the important question that remains is whether information obtained in the model based on the application of one high dose of VPA at a particular period of intrauterine development (on embryonic day 12.5th) could represent/cover the consequences of exposure to smaller doses of VPA during the entire period of intrauterine development, which replicates VPA usage in humans. An important challenge in clinical studies is to define the relationship between the VPA dose used to medicate the mother and the long-term health outcomes in offspring not suspected of having birth defects (Christensen et al., 2013; Elkjær et al., 2018; Daugaard et al., 2020).

Detailed examination of the behavioral characteristics of rodents exposed to different doses of VPA throughout intrauterine development could provide guidance for identifying the consequences specific to critical/sensitive developmental phases and separate them from those explicitly related to the VPA model of ASD. Without such an approach, it would be difficult to compare the results of this study with attempts to address VPA-induced ASD because they are the outcomes of different models. It is for this reason that the results of the present study are not extensively discussed using neurobehavioral findings of the VPA rodent model of ASD (Schneider and Przewłocki, 2005; Snow et al., 2008; Martin and Manzoni, 2014). Also, VPA-induced impairment of inhibitory and excitatory neurotransmission takes place in different brain regions, including the cortex, striatum, hippocampus, amygdala, and cerebellum (Biggs et al., 1992; Peichev, 1992; Löscher, 1993; Olexová et al., 2016; Puig-Lagunes et al., 2021; Kuo and Liu, 2022), which may be associated with a poor behavioral outcome. However, in the model of prenatal exposure to VPA, the offspring is indirectly exposed and these neurochemical findings cannot explain the behavioral characteristics.

In this study, we used a balanced approach and focused on symptoms associated with ASD that have a strong impact on the quality of life of the general population as well (Oakley et al., 2021), such as the risk for developing overweight or obesity (Dhaliwal et al., 2019), pain hyposensitivity (Klintwall et al., 2011; Yasuda et al., 2016), increased anxiety (Vasa and Mazurek, 2015), reduced “novelty bias” (Vivanti et al., 2018) and less interest in the environment (Pierce and Courchesne, 2001). The only constancy between these symptoms and the results obtained in the present study regards pain hyposensitivity in animals of both sexes (regardless of VPA dose), and increased anxiety, but only in males (and without a clear dose-response relationship), suggesting that this model replicates only a part of the associated symptoms of ASD.

The findings of this study need to be confirmed in preclinical studies using the same model and compared with upcoming clinical studies. Prenatal exposure to both low and high doses of VPA could be associated with increased anxiety-related behavior, particularly in males, while prenatal exposure to low doses of VPA could be associated with decreased anxiety-related behavior in females, leading to the conclusion that continuous prenatal exposure to VPA has its behavioral/emotional signature that is not simply a part of the natural behavioral/emotional sex-related tendencies in adolescence. Namely, adolescence is linked with difficulties in the control of behavior and emotion, thus bringing about both sensation-seeking, particularly in boys (Steinberg, 2008; Keyes et al., 2015), and internalizing of problems, particularly in girls (Kessler et al., 2012; McLaughlin and King, 2015; Ohannessian et al., 2017).

Also, the finding of the present study that continuous prenatal exposure to a wide dose range of VPA produces hyposensitivity to thermal pain in adolescent offspring has potential clinical importance. Namely, an elevated pain tolerance in adolescents has gained much attention in recent years since there are indications that self-harm is significantly associated with higher pain thresholds (Koenig et al., 2016) and that the reduced pain sensitivity evident in the response to a broad range of painful stimuli could present a phenotype of adolescents with self-harm (Cummins et al., 2021). Nevertheless, this needs a further examination of the behavior of animals in the HPT and other behavioral paradigms to get a comprehensive picture of the behavioral phenotype.

### Study limitation

This study did not address the social behavior of offspring exposed to VPA. Due to the robustness of the model and methodological approach used in the present study, this remains to be investigated. The absence of correlation between the examined parameters in different behavioral tests is a limitation. Nevertheless, as only some of the animals met the criteria set by the HPT on PND25 and the same animals were tested on PND32, we did not obtain a nociceptive response for all offspring. One group (VPA-200) did not pass the OFT. Therefore, for a correlation between the parameters to be possible, a different methodological approach is needed.

## Conclusion

This is the first study examining the influence of prenatal exposure to a wide range of doses of VPA on the physical and behavioral characteristics of offspring in adolescence in a mouse model. By focusing on the symptoms that could have a strong impact on the quality of life in the general population, this study showed that continuous intrauterine exposure to VPA results in a dose-related decline in BW in adolescent animals of both sexes, and dose-specific behavioral outcomes within sexes in terms of a supraspinal reflex response to painful thermal stimulus (but without an influence on stimulus learning), anxiety-like and risk-assessment behavior and novelty-induced psychomotor activity. The study highlights that BW and behavioral consequences of continuous intrauterine exposure to VPA are delicate for assessment in adolescent offspring since they are dose-related within sexes. It strongly emphasizes that there should be no equation between the terms “low dose” and “safe dose” in regard to the use of VPA in pregnancy. Further biochemical analyses are needed to better characterize the reported findings.

## Data availability statement

The original contributions presented in this study are included in the article/supplementary material, further inquiries can be directed to the corresponding author.

## Ethics statement

The animal study was reviewed and approved by Ethical Committee for the Use of Laboratory Animals of the Institute for Biological Research, University of Belgrade.

## Author contributions

JP contributed to the conceptualization, methodology, investigation, and writing of the original draft. SS contributed to the conceptualization and resources. BP contributed to the formal analysis, visualization, and writing of the original draft. GS and LM contributed to the investigation. VP contributed to the supervision and manuscript revision to its final form. All authors have read and approved the final version of this article.
